# Editorial: Cell Death and Targeted Cancer Therapies

**DOI:** 10.3389/fcell.2022.967720

**Published:** 2022-07-06

**Authors:** Tugba Bagci-Onder, Ozgur Kutuk, Triona Ni Chonghaile, Uwe Knippschild

**Affiliations:** ^1^ Koç University School of Medicine, Istanbul, Turkey; ^2^ Department of Immunology, School of Medicine, Başkent University, Ankara, Turkey; ^3^ Department of Physiology and Medical Physics, Royal College of Surgeons in Ireland, Dublin, Ireland; ^4^ Department of General and Visceral Surgery, Surgery Center, Ulm University Medical Center, Ulm, Germany

**Keywords:** cell death, cancer, therapy resistance, apoptosis, ferroptosis, pyroptosis

Cell death is critical for organismal development, maintenance of tissue homeostasis and the prevention of diseases, such as cancer. The most well-established mechanism of cell death is apoptosis, and cancer cells are known to adopt mechanisms to evade it. However, the roles of non-apoptotic cell death mechanisms, such as pyroptosis, necroptosis, and ferroptosis, in regulating cancer progression and therapy response are emerging. Development of therapy resistance involves the de-regulation of these cell death programs as well as tumor cell-microenvironment interactions. Our understanding of the precise mechanisms of cell death signaling networks in response to cancer therapies continue to evolve.

Resistance to therapy poses a prominent problem for the successful treatment of cancer, and there is a vast amount of information emerging in the field of therapy resistance. One of the important players in regulating resistance to therapies is the tumor microenvironment. For example, the bone marrow microenvironment can provide survival signals to leukemic stem cells, enabling survival of cells leading eventually to relapse of the disease. O’Reilly et al. developed an *in vitro* model system to study quiescent acute myelogenous leukemia (AML) cells in the bone marrow microenvironment. Using a hydrogel co-culture system, they induced quiescence in AML cells, which was enhanced in hypoxic conditions. Next, they screened a total of 1,600 drugs to identify therapeutics that could activate quiescent AML cells. Sequential treatments with hedgehog inhibitors, adipocyte peptides and tyrosine kinase inhibitors could move a proportion of cells into a cycling state. It was not clear from the studies if this also enhanced apoptosis within cells. Potentially, this could be a useful strategy to target residual quiescent cells in the bone marrow to prevent disease relapse.

Tumor treatment responses are also likely limited by layers of immune suppression in the tumor microenvironment. Yun et al. showed that the immune modulator cytotoxic T-lymphocyte-associated protein 4 (CTLA-4) had an interesting cytoplasmic role. Through anchoring phosphatase PP2A in the cytosol it led to activation of ataxia-telangiectasia mutated (ATM), thereby enhancing the DNA damage response induced by the antibiotic zeocin. This raises a hypothesis on whether CTLA-4 inhibitors could potentially have dual function in protecting T-cells from DNA damage and enhancing immune function–this however was not explored in this study and would require further validation. Next, Hagelund and Trauzold investigated the effect of the microenvironmental pH may have on the death receptor agonist TRAIL in pancreatic cancer. They found that adaptation to changes in extracellular pH could alter the response to TRAIL, with acidic pH increasing TRAIL sensitivity in one of the pancreatic cell lines. However, it is not clear how the extracellular pH could be manipulated in a therapeutic manner in the clinic. Cevatemre et al. investigated the link between metabolism and epithelial to mesenchymal transition (EMT) in the lung cancer cell line A549. Inhibition of pyruvate dehydrogenase through small molecule inhibitors or genetic approaches confers resistance to cytotoxic agents and showed phenotypic evidence of EMT. Together, these studies show that the microenvironment can be modelled and provide survival signals to the tumor, blocking cell death.

Brain tumors, especially glioblastoma, show a high resistance to standard therapies. Therefore, Burster et al. critically discuss the potential of new therapeutic concepts in their review. To activate apoptotic processes in glioblastoma cells, dual inactivation of BCL-2/BCL-xL, and MCL-1 seems to be necessary. However, drawbacks in an appropriate therapy development to induce apoptotic processes require still the establishment of new combinatorial inhibition approaches. Furthermore, new therapeutic approaches using small molecule inhibitors, oncolytic viruses, immunomodulators, or immunotherapy including peptide and mRNA-based vaccines have shown promising antitumor effects. However, only a few patients responded to immunotherapy. To improve immunotherapy concepts, it is therefore important, on the one hand, to elucidate the underlying pathophysiological mechanisms of glioblastoma and, on the other hand, to sensitize glioblastoma to immunotherapies by personalized therapy using a combination of drugs.

Since initial therapy for medulloblastoma, the most common brain tumor in children, is associated with severe side effects, research for new agents is also ongoing for this tumor entity. In this context, Dwucet et al. demonstrated that the imirilone derivate OBC201/TIC10 inhibits the growth of medulloblastoma cell lines independently of their *c-myc* status and shows synergistic effects in combination with the glycolysis inhibitor 2-deoxyglucose. Since both substances can pass the blood-brain barrier, the combined treatment of medulloblastoma patients seems to be promising approach.

Since low-dose radiation significantly increases the sensitivity of tumor cells, while protecting normal tissues at the same total dose compared with conventional fractionated radiotherapy due to the hyper-radiosensitivity/induced radioresistance (HRS/IRR) phenomenon, Wang et al. analyzed the underlying mechanism in lung cancer cells. Their results show that ATM can modulate autophagy, which is involved in regulating the hypersensitivity of lung cancer cells to low-dose radiation, via phospho c-Jun N-terminal kinase (p-JNK), and autophagy and Beclin 1 Regulator 1 (AMBRA1).

Hematological malignancies define a collective term for the heterogenous neoplastic diseases of the hematopoietic and lymphoid tissues. These disorders clinically manifest as leukemia, lymphoma or myeloma, accounting for more than 1.3 million new cases worldwide. While several treatment options including targeted therapies, monoclonal antibodies, and CAR-T cells significantly improved survival of patients with hematological malignancies, development of resistance to therapies is a prominent drawback, leading to relapse and treatment failure. Liang et al. utilized state-of-the-art data mining and network pharmacology strategies to identify common targets of a novel histone deacetylase inhibitor (chidamide) and ASA (acetylsalicylic acid, Aspirin) in AML-myelodysplastic syndrome cells. They demonstrated that combination of chidamide and ASA led to mitochondrial apoptosis in AML-myelodysplastic cells by inhibiting PI3K/AKT pathway. In addition, treatment of cells with chidamide plus ASA resulted in accumulation of cells in G0/G1 because of cell cycle arrest through downregulation of CDK4 and CDK2 and upregulation of p21 expression. Zhu et al. demonstrated that circular RNA circADD2 with a potential miR-149-5p binding site was downregulated in acute lymphoblastic leukemia (ALL) cell lines and bone marrow samples of children with ALL. Accordingly, they showed that circADD2 overexpression could trigger apoptosis in ALL through sponging miR-149-5p and reducing AKT2 expression. Of note, direct targeting of AKT2 by circADD2 remains to be validated. In another elegant study, Manzano-Munoz et al. used dynamic BH3 profiling (DBP) to identify MEK inhibitor trametinib in NALM-6 B-cell precursor acute lymphoblastic leukemia (BCP-ALL) cell line with a mutation in NRAS. Furthermore, DBP testing identified trametinib plus sunitinib (multi-target tyrosine kinase inhibitor) in SEM BCP-ALL cell line overexpressing FLT3. Importantly, coimmunoprecipitation experiments showed that trametinib led to increased MCL-1 expression to sequester BH3-only protein BIM and sunitinib promoted neutralization of BIM by BCL-2 and MCL-1 when used at lower concentrations. Targeting MCL-1 and BCL-2 by using BH3 mimetics S63845 (MCL-1 inhibitor) and venetoclax (BCL-2 inhibitor) restored sensitivity to trametinib and sunitinib in BCP-ALL cell lines, accordingly. Together, these studies explored distinct cell signaling pathways to pinpoint prosurvival signals in hematological malignancies, which may pave the way for novel combinatorial treatment options.

Non-apoptotic cell death mechanisms such as necroptosis or pyroptosis, which evoke inflammatory response, can actively participate in cancer progression. Zhang et al. performed in-depth statistical analysis of The Cancer Genome Atlas (TCGA) database and provided a pyroptosis-related gene (PRG) signature with predictive power for the clinical outcomes in endometrial cancer (EC), a highly common and aggressive gynecological tumor. Specifically, the prognostic PRG panel included ELANE, GPX4, GSDMS, and TIRAP, whose expressions were differentially regulated between EC and normal endometrial tissue. Cao et al. Similarly, demonstrated a prognostic PRG signature for uveal melanoma (UVM). In this study, authors stratified the patients into high-risk and low-risk groups and identified PRGs associated with each group. ANO6, CEBPB, and TMEM173 were upregulated, and VIM and TXNIP were downregulated in the high-risk group. Authors further examined the role of one of these PRGs, ANO6, via functional assays in UVM cell culture and showed that ANO6 silencing led to attenuation of cell migration. Pyroptosis is mainly an inflammatory cell death mechanism and involves the perforation of cell membrane and release of cellular contents. Its de-regulation is not exclusive to cancer, but also observed in several other diseases, such as the Osteoarthritis (OA). Yang et al. Compiled a large body of evidence to demonstrate the relation of pyroptotic, apoptotic, necroptotic and ferroptotic cell death mechanisms to OA. Accordingly, understanding these links will provide the design of successful treatment strategies. Finally, ferroptosis is an alternative cell death mechanism characterized by iron accumulation and lipid peroxidation. Yan et al. provided a ferroptosis-related gene signature composed of 6 genes (CISD1, CRYAB, FTH1, ACACA, ZEB1, and SQLE) with prognostic value in bladder cancer (BC), using TCGA and Gene Expression Omnibus databases. Authors constructed a pedictive risk model that involved ferroptosis-related genes and found relevance to the EMT status, immune response and mutation status of key BC-related genes. Tang et al. comprehensively reviewed the mechanisms of ferroptosis, its relation to cancer and provided future perspectives for cancer therapy. Accordingly, induction of ferroptosis using single agents or combination therapies with immunotherapy, radiotherapy, chemotherapy, or photodynamic therapy may be successful anti-cancer approaches. However further research is necessary for clinical translation. Combined, these studies show the complexities of tumor resistance to standard and targeted treatments. Novel combinatorial approaches were identified that may pave the way to improved treatment outcomes.

